# Upright and supine assessment of pelvic floor muscle defects in women with and without prolapse

**DOI:** 10.1038/s41598-026-35598-z

**Published:** 2026-01-17

**Authors:** Irina de Alba Alvarez, Frieda van den Noort, Frank F.J. Simonis, Anique T.M. Grob

**Affiliations:** 1 Multi Modality Medical Imaging (M3I) TechMed Centre , University of Twente , The Netherlands, Enschede, Netherlands; 2 Department of Gynecology, Ziekenhuisgroep Twente, Hengelo, Netherlands

**Keywords:** Pelvic organ prolapse, Pubococcygeus, Iliococcygeus, Iliococcygeus angle, Anatomy, Diseases, Health care, Medical research

## Abstract

The levator ani muscle (LAM), crucial for pelvic organ support, can develop defects that contribute to pelvic floor disorders (PFD) like pelvic organ prolapse (POP). Factors such as childbirth, aging, and menopause increase the risk of LAM damage. While MRI is commonly used to assess LAM, most studies use the supine position, potentially underestimating POP severity. Upright MRI offers a more accurate view of pelvic floor support under natural conditions. The aim of this study was to compare the pelvic floor support across four groups of women—nulliparous, parous premenopausal, parous postmenopausal, and women with POP, by analysing pubococcygeus muscle (PCM) and iliococcygeus muscle (ICM) defects and the iliococcygeus angle (ICA) using a tiltable magnetic resonance imaging (MRI) scanner. Sixty-three women were included and divided into 4 groups: 15 nulliparous (Nulli), 15 parous-premenopausal (Par-pre), 15 parous-postmenopausal (Par-post) and 18 patients with minimum stage 2 prolapse of the anterior vaginal wall or uterus (POP). Women underwent supine and upright MRI scans. PCM and ICM defects were assessed on both the supine and upright scans. Defects were categorized as “none”, “minor” or “major” following previously established guidelines. Using upright MRI scans and manual segmentation of the LAM, the ICA was determined in 5planes from anterior to posterior. Statistical testing was performed using one-way ANOVA and Bonferroni post-hoc test. The results show that nulliparous women had no PCM defects. Par-pre, Par-post and POP groups showed varying rates of major PCM defects (7.7%, 16.7%, and 68.8% respectively). Minor ICM defects were present in all groups (21.4% (Nulli), 61.5% (Par-pre), 66.7% (Par-post), and 55.3% (POP)), but only the POP group had major defects (43.8%). The ICA was significantly sharper (*p* < 0.001) in the POP group compared to other groups. A decrease in pelvic floor support, assessed by PCM damage in supine and ICM damage and a sharper total ICA done in upright, was measured in the POP population, as compared to healthy women. Minor muscle defects were common across all groups, but major defects exclusively in POP patients. This fundamental insight on muscle quality adds to our anatomical knowledge on the occurrence of POP.

## Introduction

The levator ani muscle (LAM) is a group of muscles that plays a crucial role in supporting the pelvic organs. It consists of three distinct muscles: the pubococcygeus muscle (PCM), the iliococcygeus muscle (ICM), and the puborectalis muscle (PRM)^1^. Defects such a as ballooning with or without muscle thinning, a diffuse muscle shape (small gaps), focal bulging or hernias, in the LAM can lead to a variety of pelvic floor disorders (PFD), including urinary incontinence (UI), faecal incontinence, and pelvic organ prolapse (POP)^2–7^. LAM abnormalities are reported for 20% of women after vaginal delivery^8^, with aging and menopause as additional risk factors which contribute to the weakening of the pelvic floor and increase the risk of POP^9,10^.

Assessment of the LAM in general, and POP in particular, can be done by using medical imaging techniques, such as Magnetic Resonance Imaging (MRI). MRI assessment enables the evaluation of the pelvic floor’s supportive structures. This assessment can be objectified by the application of previously published muscle defect assessment protocols for the ICM^11^ and PCM^12^.

A key feature of the LAM support is the iliococcygeus angle (ICA)^13–16^,. The ICA is the angle in coronal plane formed by the ICM and the transverse plane of the pelvis^15^. Women with POP are reported to have a significantly larger ICA than nulliparous women (57.95° compared to 33.4°)^13^ during straining, which is thought to reflect a decrease in LAM support^13,17^. Most MRI studies on assessing POP and LAM support are conducted with the patient in supine position. However, patients experience their PFD complaints mainly when in upright position and various studies have shown that POP is underestimated on supine MRI^11,18,19^. Upright MRI makes it possible to image and assess the pelvic floor in the position when the weight of the organs is above the LAM and when patients experience their symptoms.

To provide new fundamental insights in the differences between supine and upright position with respect to the pelvic floor support, this study compares morphological characteristics of the pelvic floor in women with different age and parity using MRI in supine and upright position. Specifically, we aim to determine differences in the female pelvic floor anatomy in women with and without POP based on the ICA and defects in the PCM and ICM using two dedicated protocols^11,12^.

## Method

The study was conducted with 63 women over 18 years of age, divided into four groups according to the following inclusion criteria:

- Nulli: 15 women with no previous history of vaginal delivery (nulliparous), pre-menopausal status, no symptoms related to POP or incontinence.
- Par-pre: 15 women with previous history of at least one vaginal delivery (parous), pre-menopausal status, no symptoms related to POP or incontinence.
- Par-post: 15 women with previous history of at least one vaginal delivery (parous), post-menopausal status, no symptoms related to POP or incontinence.
- POP: 18 women with previous history of at least one vaginal delivery (parous), symptomatic prolapse, with pelvic organ prolapse quantification (POP-Q) stage ≥ 2^20^.

POP patients were included from the gynaecology department of the local hospital. The 45 asymptomatic women (groups “Nulli”, “Par-pre” and “Par-post”), were recruited as controls via flyers. The study was approved by the medical ethics committee (CMO Regio Arnhem Nijmegen NL74061.091.20). The methods were performed in accordance with the guidelines and regulations of the ethics committee. Written informed consent was obtained from all participants.

### Exclusion criteria

Women were excluded if they were unable to stand upright for 20 min without assistance due to the time required to acquire the standing MR scans; failed to meet the MR safety checklist requirements for undergoing an MR scan; or if they had a jeans size of ≥ 52(EU) or ≥ 22(US), due to the restricted coil circumference.

### MR imaging

All participants were asked to empty their bladder 15 min before the scan. MRI was performed using a tiltable 0.25 T MR scanner (G-Scan Brio, Esaote, Genoa, Italy). All patients were scanned using a multichannel spine coil in upright position (81 degrees rotation with respect to horizontal position) and in supine position. Static images of the pelvis were acquired using the 3D balanced steady state free precession (bSSFP) sequence (spatial resolution 0.49 × 0.49 × 0.49 mm³, echo time [TE] = 4 ms, repetition time [TR] = 8 ms, flip angle of 60°, field of view of 250 × 250 × 122 mm³ and acquisition matrix of 124 × 124 × 76. However, after several patients this was enlarged to 250 × 250 × 160 mm³ and 124 × 124 × 100 to also include the ischial spines.

### Defect evaluation

**Fig. 1:**
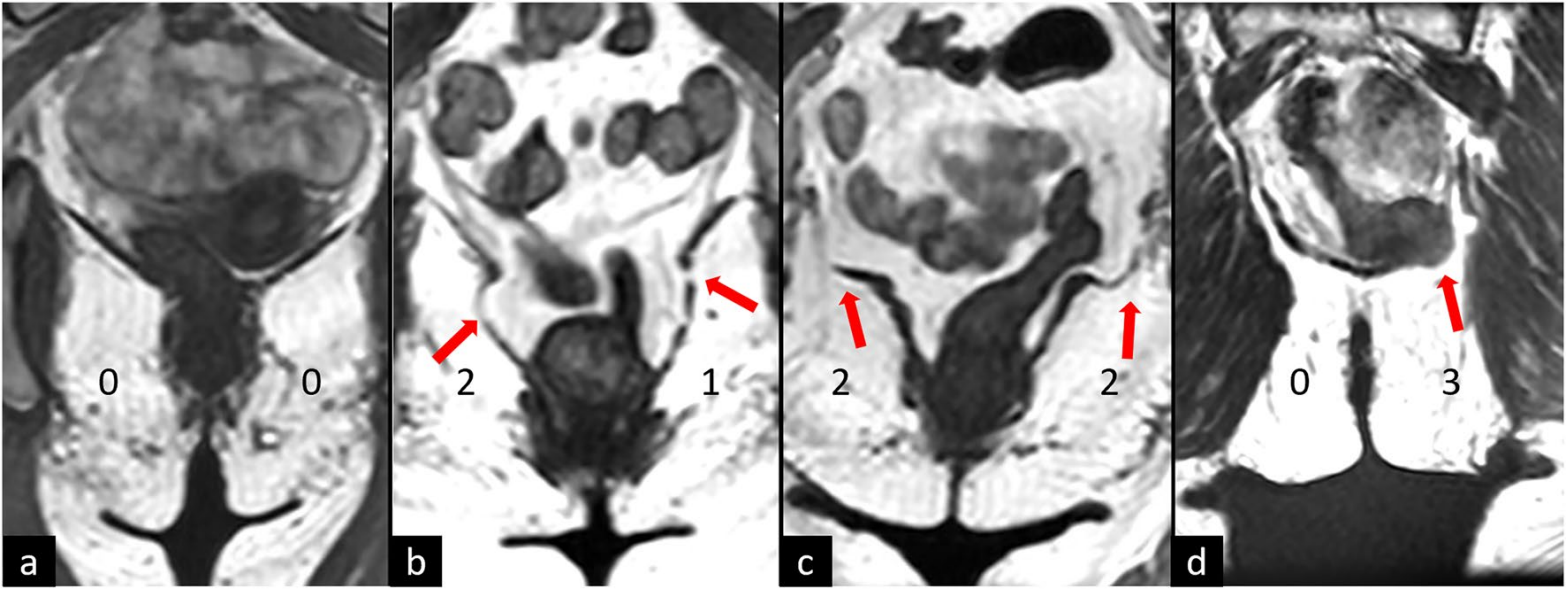
Cropped coronal images of the iliococcygeus muscle (ICM) at the level of the mid-rectum. **a)** example of ICM with no defects. **b**) Example of woman with minor defect, right side (score 2) showing thinning and bulging and left side (score 1) showing gaps and generalized bulging. **C**) Example of major defect in ICM where focal bulging and thinning can be seen in both sides of the muscle. d) Major defect in the ICM of herniation with rectal protrusion. Red arrows illustrate points of muscle defects.

The evaluation of MR images involved a careful assessment of the LAM defects. These defects were categorized based on their location as either PCM or ICM defects^15^. In our study, we employed two muscle evaluation protocols to assess muscle integrity, each utilizing a scoring system based on the severity of observed abnormalities. Under the protocol developed by Kearney et al.^12^, the right and left sides of the PCM were separately evaluated in supine position and assigned scores ranging from 0 to 3, indicating the extent of abnormalities observed. A total score was derived by summing the scores from both sides, with categorization into normal or no damage (0), minor damage (1–3), and major damage (4–6). Importantly, if either side received a score of 3, the total score was automatically categorized as major damage. The evaluation of the ICM was done in upright position with a protocol previously established^11^. Similar to the PCM protocol, the ICM protocol evaluated the left and right sides separately, with scores ranging from 0 to 3 and the total score was determined and categorized accordingly. Notably, if either side presented with a hernia (score of 3), the total score was automatically classified as major damage (Fig. 1). Following the results of a previous study^11^, indicating poor visibility of the PCM in supine position and significant underestimation of ICM damage in supine position, the PCM defects were evaluated solely in supine position, and ICM defects solely in upright position.

### ICA data processing

Upright images were processed using 3D Slicer 5.0.3^21^. The initial step involved the manual 3D segmentation of the LAM. Subsequently, four anatomical reference points (pubic symphysis, sacrococcygeal joint, right ischial spine and left ischial spine) were identified. These landmarks were used to standardize the position of the segmentations for further calculations using the 3D Pelvic Inclination Correction System (PICS)^22,23^ Data was processed using MATLAB (R2022b)^24^.

**Fig. 2:**
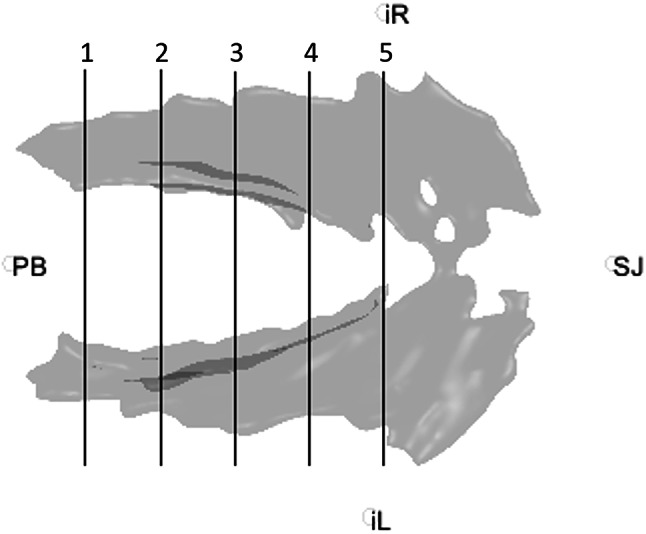
Top view of a three-dimensional model of the levator ani showing the five equidistantly defined planes. Each plane corresponds to one fifth of the distance between the position of the pubic symphysis and the ischial spines. *PB pubic bone*; *iR*,* right ischial spine; iL*,* left ischial spine; SJ*,* sacrococcygeal joint.*

**Fig. 3:**
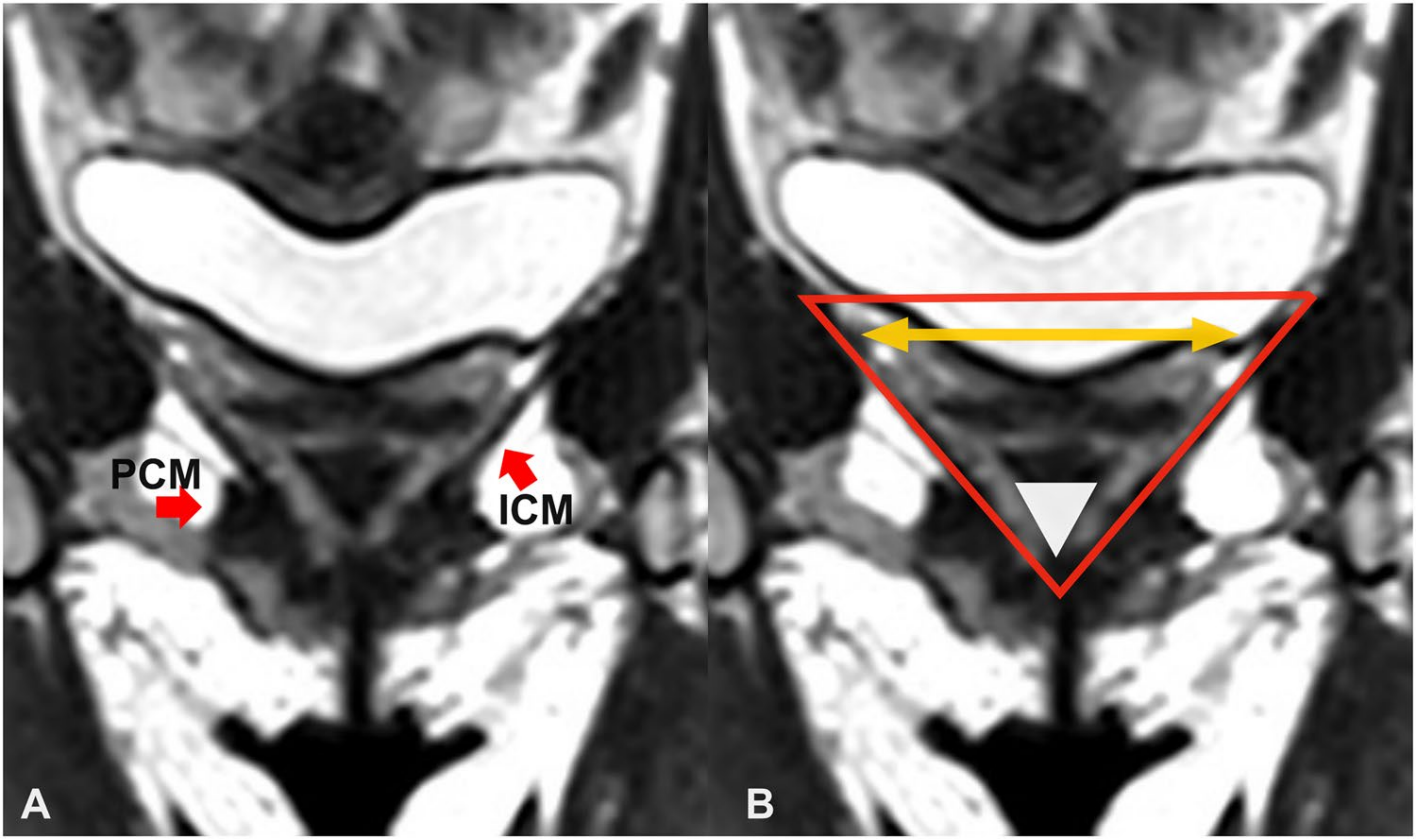
Coronal view of LAM. (A) The pubococcygeus muscle (PCM) and iliococcygeus muscle (ICM) are shown with the red arrows. B) The yellow arrows show the right and left iliococcygeal angle (ICA). Considering LAM as a triangular shape the total ICA can be seen at the white arrowhead.

The LAM segmentation was divided into 5 equidistant planes from the pubic symphysis to the right ischial spine. Each of these planes is perpendicular to the PICS line and correspond to the position where ICAs were measured (see Fig. 2). The right and left ICA were determined using a first-degree polynomial fit through the slice of LAM segmentation in each of the planes. The left and right angle of the ICA were determine, from which the total ICA was obtained (based on a triangle shape) using this formula: total ICA = 180 – left ICA – right ICA (Fig. 3). In contrast to previous literature, we report both the left and right, as well as the a total ICA, since the total ICA reflects the support of the ICM with one single measurement, with a larger ICA representing more pelvic support by the LAM.

Data were analysed using IBM SPSS Statistics (Version 28.0.1.0(142)). The Shapiro-Wilk test was used to check for a normal distribution, significant differences in the total ICA between the four groups were tested using one-way ANOVA. Bonferroni post hoc test^25^ was used for multiple comparisons of the groups. In case of a detachment or avulsion of the LAM from the pubic bone, this can cause an enlarged gap between the two in plane 1 (Fig. 2). If the angle could not be measured within the segment corresponding to a plane, that particular plane would be omitted from statistical analysis.

## Results

From the 63 women included in the study, 8 women were excluded for further analysis due to: insufficient image quality caused by bowel movement (*n* = 1, Nulli group), lack of ischial spine identification (*n* = 5 women (*n* = 2 Par-pre, *n* = 1 Par-post and *n* = 2 POP)) and reported de novo POP symptoms within one week after the MR scan (*n* = 2 Par-post group). Leading to a total of 55 women (14 nulliparous, 13 parous pre-menopausal, 12 parous-postmenopausal and 16 POP) for analysis. Demographics per group are listed in Table 1.

**Table 1:** Demographic data of the study population.

	Total (*n* = 55)	Nulli (*n* = 14)	Par-pre (*n* = 13)	Par–post (*n* = 12)	POP (*n* = 16)
Age (years)	48 ± 18	23 ± 2	40 ± 6	58 ± 4	67 ± 7
BMI (kg/m ^2^)	26 ± 4	22 ± 2	26 ± 5	26 ± 3	28 ± 4
Parity	2 ± 1	0 ± 0	2 ± 1	2 ± 1	2 ± 1

Values are mean ± SD. BMI, body mass index.

### PCM defects (supine)

**Fig. 4:**
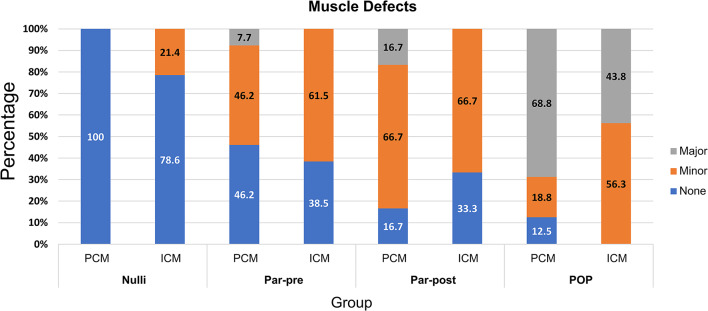
Percentages of none (blue), minor (orange) and major (grey) defects in the pubococcygeus (PCM) and iliococcygeus (ICM) in the nulliparous group (Nulli), the parous premenopausal group (Par-pre), the parous postmenopausal group (Par-post) and the group of women with pelvic organ prolapse (POP).

**Fig. 5:**
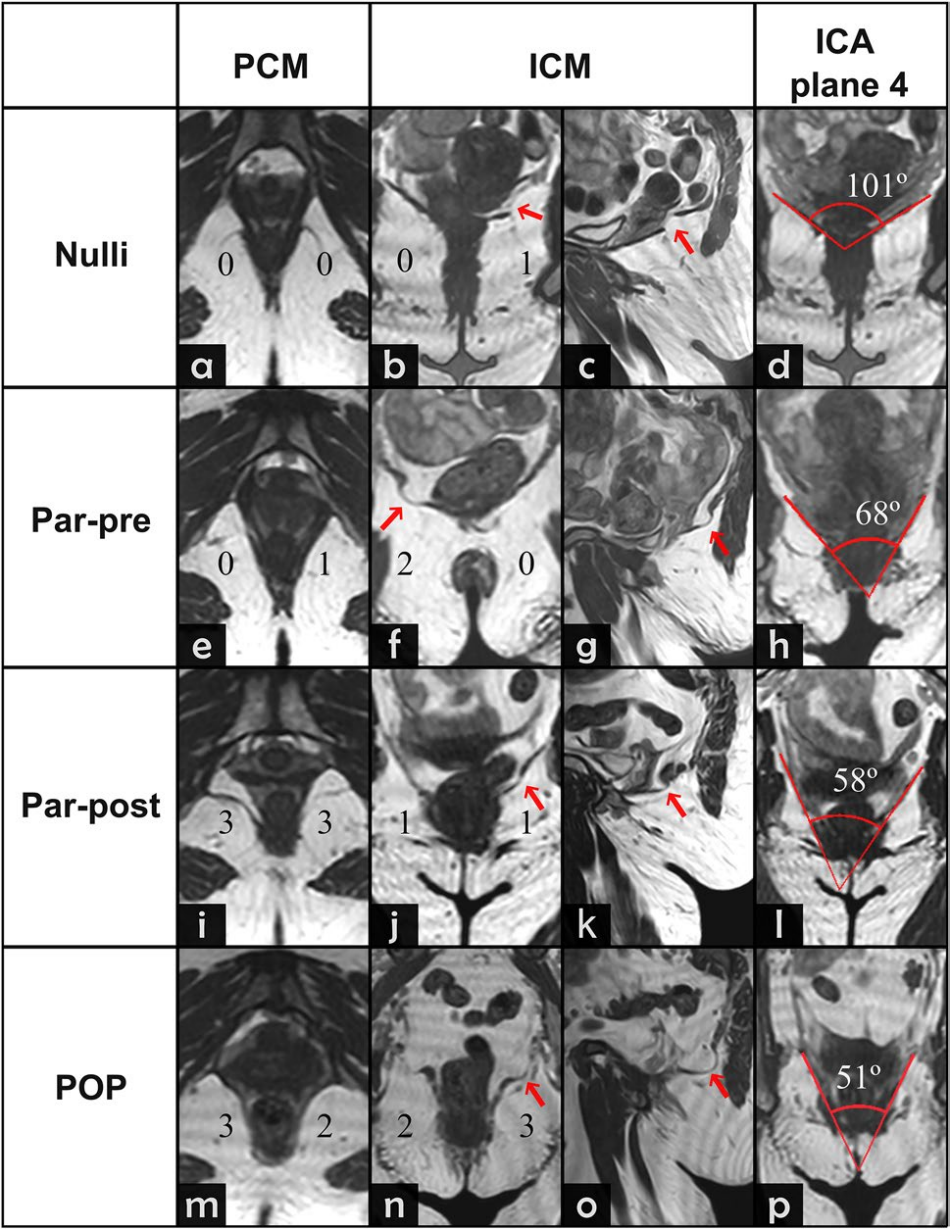
Examples of different degrees (0, 1, 2 or 3) of defects in the pubococcygeus muscle (PCM) using supine MRI and in the iliococcygeus muscle (ICM) and total iliococcygeus angle (ICA) in plane 4 using upright MRI, in the four populations. Images (**a-d**) correspond to a nulliparous woman (Nulli), there are no PCM defects (**a**), but there is a larger gap in the left ICM visible in coronal (**b**) and sagittal (**c**) view, which is considered minor ICM defect; in (**d**) the ICA shows and obtuse value. Images (**e-h**) correspond to a parous-premenopausal woman (Par-pre), there is a minor unilateral defect in the PCM (**e**), a unliteral bulging in the right ICM can also be seen in the coronal and sagittal images (**f**,** g**) scoring minor defect in both muscles; in (**h**) the ICA shows a sharper value than Nulli group. Images (**i-l**) correspond to a parous-postmenopausal woman, in the axial images (**i**) major defects can be seen in the PCM where the vaginal wall in both sides is protruding close to the internal obturator muscle, there is also visible a thinning of the ICM in the coronal and axial (**j**,** k**) images; image (l)shows the ICA, sharper than the Par-pre group. Images (**m-p**) correspond to a patient with pelvic organ prolapse (POP), the axial image (**m**) shows the PCM with a major defect, there is thinning of the right ICM and a herniation in the right ICM visible and coronal (**n**) and the herniation is visible in sagittal (**o**), scoring a major defect.; it is visible in image (**p**) the POP group presents the sharpest values in the ICA.

The prevalence and severity of PCM defects differed between groups (Fig. 4). No PCM defects were observed in the Nulli group (Fig. 5a). In contrast, minor PCM defects were present in 46.2% of women in the Par-pre group (Fig. 5e), 66.7% in Par-post group and 18.8% of the women in the POP group. Major PCM defects were present in one woman (7.7%) of the Par-pre group and two women (16.7%) of the Par-post group (Fig. 5i). In the POP group 11 women (68.8%) exhibited major PCM defects (Fig. 5m).

### ICM defects (upright)

The ICM defects identified in upright differ between the four groups (Fig. 4). Minor defects were found in 21.4% of the women (*n* = 3) in the Nulli group (Fig. 5b and c), while minor defects occurred in 61.5% and 66.7% of the women in the Par-pre (Fig. 5f and g) and Par-post (Fig. 5j and k) groups respectively. No major defects were found in any of these non-POP women, in all POP patients had minor (56.3%) or major (43.8%) defects in their ICM and three of them presented hernias (Fig. 5n and o).

### Total ICA

LAM detachment or avulsion was found in one woman from the Par-pre group, five women from the Par-post group, and three patients from the POP group. As a result, ICA calculations in plane 1 was not feasible and therefore the first plane was excluded from statistical analysis.

**Fig. 6:**
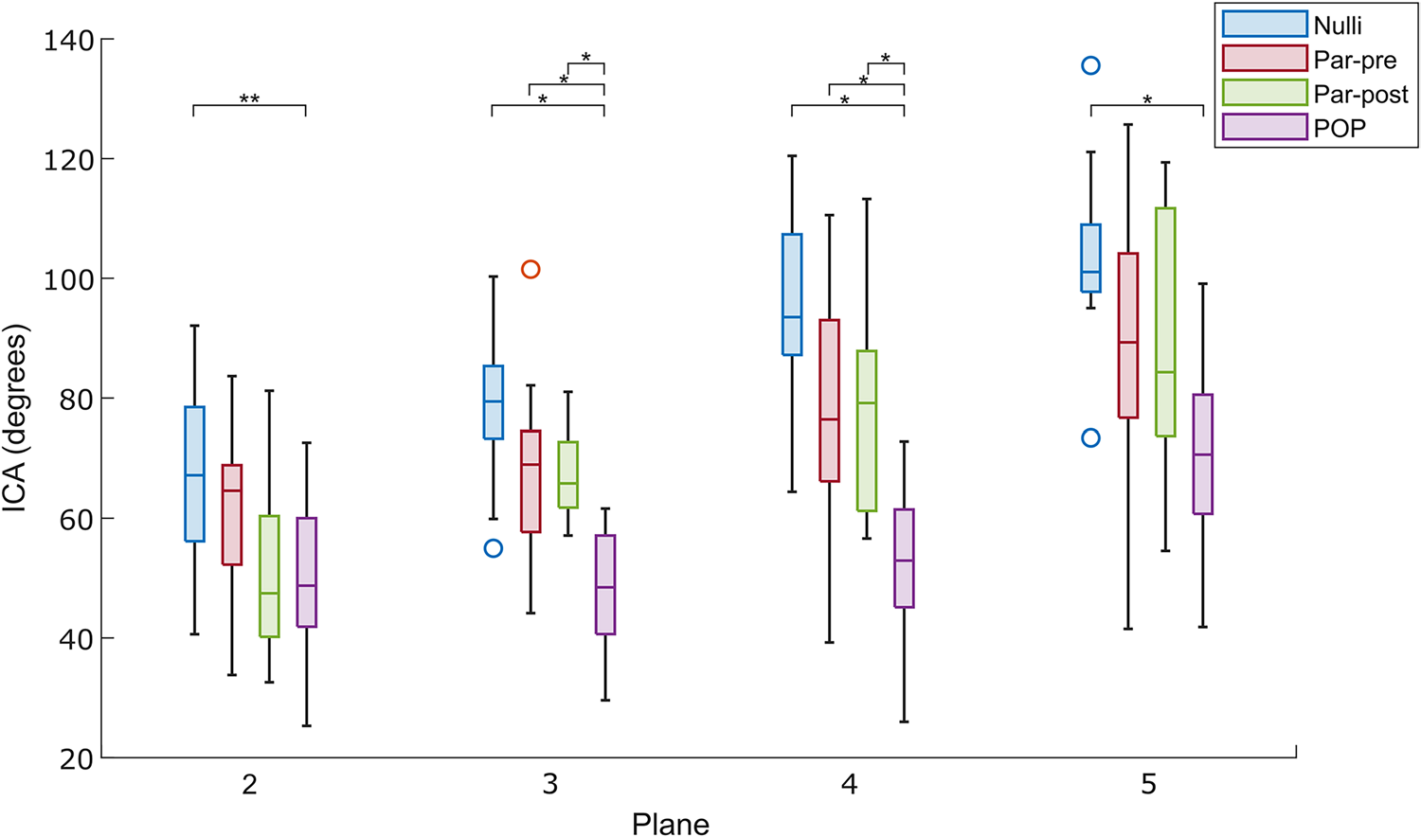
Boxplots (median and interquartile range (IQR) of the Iliococcygeus angle (ICA) values of the 4 populations. Nulliparous group (Nulli), parous premenopausal group (Par-pre), parous postmenopausal group (Par-post) and patients with pelvic organ prolapse (POP) on planes 2, 3, 4, 5 are illustrated. Significant differences between groups are visualized on top, *p* < 0.001 (*), *p* = 0.02 (**). A significant larger ICA is found in all non-POP groups in planes 3 and 4 as compared to the ICA of the POP group. In planes 2 and 5 the ICA is solely significantly larger in the Nulli group as compared to the POP group.

The Shapiro-Wilk test was applied to the four groups in each of the five planes for the total ICA and showed normal distribution. Applying a one-way ANOVA revealed a statistically significant difference in ICA between groups in plane 2, 3, 4 and 5, with p-values of 0.016, < 0.001, < 0.001 and < 0.001 respectively. The results of the Bonferroni post hoc test revealed significant findings across different planes and measures between healthy populations and patients with POP (Fig. 6). The results of each population and total, right and left ICA per plane are included in Table 2.

**Table 2:** Mean total ICA (SD) [Left ICA I right ICA].

Planes	2	3	4	5
Nulli	66 (± 15) [58 I 56.0]	78 (± 12) [52 I 50]	95 (± 15) [43 I 42]	104 (± 15) [39 I 38]
Parous	61 (± 16) [61 I 58]	69 (± 15) [57 I 54]	79 (± 21) [53 I 49]	88 (± 23) [47 I 45]
Meno	52 (± 16) [66 I 62]	67 (± 8) [59 I 54]	80(± 19) [50 I 51]	88 (± 22) [47 I 45]
POP	49 (± 14) [66 I 66]	48 (± 10) [67 I 65]	53 (± 12) [64 I 63]	70 (± 16)[55 I 55]

The values in the table are angles expressed in degrees.

Main differences in ICA were found in planes 3 and 4. In both planes, POP patients showed significantly sharper total ICA compared with Nulli, Par-pre and Par-post women (all *p* < 0.001), but no significant differences were found among the three control groups.

In plane 3, mean ICA was 48.3° ± 10.2° in the POP group compared with 77.8° ± 12.4° in the Nulli group, 68.5 (± 14.7) in the Par-pre group and 67.2 (± 7.6) in the Par-post group.

In plane 4, mean ICA was 53.4° ± 11.9° in the POP group (Fig. 5p), compared with 95.2 (± 14.6)in the Nulli group (Fig. 5d), 78.6 ° ± 21.3° in the Par-pre group (Fig. 5h), and 79.7° ± 19.3° in the Par-post group (Fig. 5l).

In planes 2 and 5, total ICA differed significantly only between POP and Nulli groups. POP group exhibited a significantly sharper ICA in plane 2 (49° ± 13.7°) compared with Nulli group (65.7° ± 14.7°) (*p* = 0.02). Similarly, in plane 5, ICA was significantly sharper in POP group (70° ± 16°) than in Nulli group (103° ± 14.6°) (*p* < 0.001).

### Discussion

This study provides new fundamental insights in the differences in pelvic floor muscle integrity and support in women with and without pelvic floor disorders when assessed in upright position as compared to the supine position. All POP patients had defects in the ICM, with the exclusive presentation of major ICM defects within this group. Also, 68.8% of the POP patients exhibit major defects in the PCM. A surprising observation emerged among nulliparous women, where 21.4% of the individuals manifested minor defects in the ICM, however none displayed defects in the PCM. Finally, a significantly smaller ICA is observed in POP patients compared to the other groups, particularly in plane 3 and 4, which are at the level of the vaginal canal and the rectum, reflecting a lack in LAM support.

When assessing the damage in upright to the ICM in POP patients, we found 43.8% and 56.3% for major and minor damage respectively, a previous study of de Alba Alvarez et al.^11^ found to 51.6% and 45.5% major and minor damage. The differences observed in these findings may result from the smaller POP group in our study.

The relatively new and non-standard clinical care of upright MRI, both in clinical settings and research could limit the broader applicability and comparability of the study findings when compared to more commonly employed conventional imaging approaches. However, to optimize POP care new insights in the most relevant position are essential and need to be acquired by means of upright assessment.

A limitation of this study is the relatively small sample size within each group, which may have limited the statistical power to detect more subtle differences, particularly in the context of multiple comparisons. As a result, some potentially meaningful associations may not have reached statistical significance. Therefore, the findings should be interpreted with appropriate caution and considered exploratory. Future studies with larger cohorts are warranted to confirm these results and to further assess the robustness of the observed differences. Even though the sample size per groups were relatively small, groups were homogeneous, and results showed significant differences, showcasing the effectiveness of our approach.

Even though PCM and ICM damage assessment in POP patients has been done rather extensively^3,7,8,11,12^ a study on PCM and ICM damage in women without PFD (nulliparous and parous) is very limited. We found two studies by Cui et al.^26^ and Zhang et al.^17^, describing the absence of ICM damage in nulliparous women and the presence of LAM defects in women without PFD respectively. Differences in reported damage might be due to difference in patient position during assessment (upright as compared to supine), but sufficient data is lacking to draw definitive conclusions.

The assessment of hernias in patients with POP resulted in a total of 18.7%. This finding is in line with previous studies on hernia’s ranging from 13.5 to 15%, executed with the patients in supine straining position^27–29^. We found a larger deviation of hernia incidence as compared to de Alba Alvarez et al.^11^ where the herniation rate was 31.2%. This difference might be explained by the study population. In de Alba Alvarez et al.^11^ all women were scheduled for prolapse surgery while for this study POP patients were on pessary treatment. The hypothesis is that patients with a pessary have in general a better pelvic floor integrity and thus biased our current incidence towards a lower hernia rate.

From a methodological point of view, we decided to analyse the ICA as one total IC angle, rather than two separate (left and right) angles. This method differs from previous literature, but one total ICA directly relates to the support of the pelvic floor, with a smaller angle reflecting less and a bigger angle reflects more LAM support. The ICA results in upright illustrating a significant difference between POP patients and women without PFD^17^ and POP patients and nulliparous women^13^ are consistent with previous literature, where ICA was measured at the level of the ischial spine and during straining^13–15,17^.

The implications of these results lie within the field of future medical considerations for diagnostic accuracy, therapeutic strategies and management of POP. Our findings on LAM trauma and ICA prompt further exploration into the correlation with symptoms, pessary success rate and surgery recurrences.

It is imperative to note that nulliparous women exhibited certain anomalies in the ICM visible in upright MRI. Further investigation is needed to determine the prevalence of these abnormalities and to establish their role as either normal variation or in the development of POP symptoms.

### Summarize and conclusion

In conclusion, a lack of pelvic floor support, measured by PCM damage in supine and ICM damage and reduced ICA in upright, was measured in the POP population, as compared to women without pelvic floor disorders. Minor damage to the ICM can be found in all women, while major damage in the ICM is only present in POP patients. Continuing studying women with and without POP in upright position is necessary for a better fundamental understanding of the pelvic floor. In time, pelvic floor upright assessment could play a role in optimizing prolapse care including pessary management and surgery.

## Data Availability

The datasets used and/or analysed during the current study are available from the corresponding author on reasonable request.
